# Targeting oncogenic NTSR1 with liensinine reprograms Gq-mediated signaling to suppress lung adenocarcinoma

**DOI:** 10.1186/s13020-026-01356-6

**Published:** 2026-03-25

**Authors:** Yongfu Wang, Wei Liu, Pengzhuo Tao, Changmin Liu, Yizhen Yuan, Yajing Xue, Hanting Yang, Xiongfeng Liu, Xinyi Zhou, Shilin Chen, Chi Song

**Affiliations:** 1 School of Pharmacy, Chengdu University of Traditional Chinese Medicine, Chengdu, 611137 China; 2 Institute of Herbgenomics, Chengdu University of Traditional Chinese Medicine, Chengdu, 611137 China; 3 Hubei Shizhen Laboratory, Wuhan, 430061 China

**Keywords:** Lung adenocarcinoma, Neurotensin receptor 1, Liensinine, Gq protein signaling, Transcriptional reprogramming, Bioinformatics analysis, Targeted therapy

## Abstract

**Background:**

Lung adenocarcinoma (LUAD) is a leading cause of cancer death. Neurotensin receptor 1 (NTSR1), a G protein-coupled receptor, is overexpressed in LUAD and linked to poor prognosis, but its therapeutic potential is underexplored.

**Methods:**

This study combined multi-database bioinformatics to systematically evaluate the expression, prognostic relevance, genetic alterations, and immune-microenvironment association of NTSR1 across cancer types. The ONE-GO biosensor, SPR, PRESTO-Tango, and in-cell ELISA assays was used to confirm that liensinine specifically inhibits NTSR1-mediated G-protein signaling. Functional consequences of NTSR1 depletion and liensinine treatment—including effects on proliferation, apoptosis, and global transcription—were examined in vitro via CCK-8, flow cytometry, RNA-seq and Western blot. Key findings were validated by RT-qPCR in cellular models and further supported using LUAD patient cohorts from the UALCAN platform.

**Results:**

Bioinformatics analysis confirmed significant upregulation of NTSR1 in LUAD, and its high expression was closely associated with advanced tumor stage, remodeled immune microenvironment, and poor overall patient survival. ONE-GO profiling revealed preferential coupling of NTSR1 to Gα_q_. Liensinine bound NTSR1 with micromolar affinity and selectively inhibited NTSR1‑mediated Gq signaling while sparing β‑arrestin recruitment and agonist-induced internalization. Functional experiments demonstrated that either NTSR1 knockdown or liensinine treatment significantly inhibited A549 cell proliferation and migration while inducing apoptosis, with the effect of liensinine being dependent on NTSR1 presence. These findings were consistently validated in HCI‑H1299, HCI‑H1975, and PC‑9 cell lines. Transcriptomic analysis revealed that NTSR1 overexpression enriched pro-oncogenic pathways such as neuroactive ligand-receptor interaction and calcium signaling. In contrast, liensinine treatment reversed these alterations and shifted the transcriptional program toward pathways including steroid biosynthesis and protein processing in the endoplasmic reticulum. Western blot analysis has further confirmed that liensinine reduced the phosphorylation of PKC and ERK guided by NTS/NTSR1. Furthermore, we identified a core gene signature comprising *PTGS2*, *SPR1*, *ABCG1*, and *ABCA1*, whose expression was synergistically regulated by NTSR1 and liensinine and demonstrated prognostic value in LUAD patients.

**Conclusions:**

NTSR1 is a key oncogenic driver in LUAD. Liensinine is a novel NTSR1 antagonist that suppresses tumors by selectively inhibiting Gq signaling and reprogramming the transcriptional landscape.

## Introduction

Lung cancer remains one of the most challenging malignancies worldwide and is the leading cause of cancer-related mortality [1]. Non-small cell lung cancer (NSCLC) is the predominant histological subtype, accounting for approximately 85% of all cases, with lung adenocarcinoma (LUAD) representing the most common form. Its high incidence is linked to multiple complex etiological factors, including tobacco smoking, environmental pollution, occupational exposures, and genetic predisposition [2]. Over the past two decades, advancements in early detection and surgical techniques, together with breakthroughs in systemic therapies—notably targeted agents and immunotherapy—have substantially improved patient survival and quality of life [3]. Despite these advances, lung cancer, particularly NSCLC, continues to be a major cause of cancer-related death, imposing a substantial burden on both society and affected individuals. The clinical importance of NSCLC further stems from its pronounced heterogeneity and therapeutic complexity. The majority of patients are diagnosed at an advanced stage, precluding curative surgical intervention. Although targeted therapies have shown remarkable efficacy in patients with driver‑gene alterations such as EGFR, ALK, and ROS1, acquired resistance remains a pervasive challenge, and a considerable subset of patients still lacks actionable therapeutic targets [4–6]. Meanwhile, immune checkpoint inhibitors have provided durable clinical benefits for some patients [7, 8]; however, their overall response rates are modest, and robust biomarkers to reliably identify responding populations are currently lacking [9]. Moreover, treatment‑related toxicities, high economic costs, and patients’ growing expectations for quality of life continue to pose ongoing challenges to existing treatment paradigms. Therefore, there is a pressing need to explore novel therapeutic strategies, identify new druggable targets, and develop innovative pharmacological agents.

G protein-coupled receptors (GPCRs), the largest membrane receptors family encoded by the human genome, are central regulators of cellular signal transduction and diverse processes such as proliferation, survival, migration, metabolism, and immune evasion [10]. They represent one of the most successful classes of drug targets [11]. As of the latest assessments, a total of 337 drugs targeting 133 distinct GPCRs are undergoing clinical trials across all therapeutic areas, with a growing emphasis on oncology and immunology in recent years [12, 13]. Notably, nearly 70 GPCR-targeting agents are currently in clinical development for cancer treatment, including both repurposed approved drugs and novel therapeutics such as ligand modulators, antibodies, chemokine receptor antagonists, and peptide-based drugs [12, 13]. These figures remain dynamic and may shift as trials progress.

Among the numerous GPCR members, the neurotensin receptor 1 (NTSR1) has garnered considerable attention due to its notable cancer-promoting roles in various malignancies [14, 15]. Its endogenous ligand, neurotensin (NTS), is a 13-amino-acid peptide hormone widely distributed in the central and peripheral nervous systems [16]. Beyond its regulatory functions in the endocrine and paracrine activities of the digestive system, NTS exhibits growth factor-like properties in various tissues, promoting cell proliferation and survival [17, 18]. Mechanistically, upon NTS binding, NTSR1 primarily couples to Gα_q/11_ proteins, activating phospholipase C (PLC). PLC catalyzes the hydrolysis of PIP2 into IP3 and DAG, subsequently triggering intracellular calcium release and protein kinase C (PKC) activation [19]. PKC can directly phosphorylate RAF-1 in a Ras-independent manner, activating the MAPK/ERK signaling pathway. This cascade enhances the expression of transcription factors such as AP-1, NF-κB, Egr-1, Elk-1, and c-Myc, forming a molecular network that fosters cell proliferation, migration, invasion, and anti-apoptosis. Furthermore, PKC can activate protein kinase D (PKD), further amplifying NF-κB-mediated inflammatory and pro-tumorigenic signals [20, 21]. NTSR1 expression is significantly elevated in a variety of solid tumors, including gastric, pancreatic, colorectal, and prostate cancers, LUAD, and triple-negative breast cancer [14, 22–26]. Its expression correlates strongly with advanced TNM stage, lymph node metastasis, distant metastasis, and poor overall survival in patients. Notably, in LUAD, high NTSR1 expression is closely associated with resistance to EGFR-tyrosine kinase inhibitors (TKIs), remodeling of the tumor immune microenvironment (TIME), and unfavorable prognosis, suggesting its potential utility as a prognostic biomarker and a therapeutic target [27].

This study performed a comprehensive multi‑database analysis to delineate the expression profile, prognostic relevance, genetic alterations, methylation status, and immune microenvironment association of NTSR1, identified a novel small-molecule antagonist, liensinine, and confirmed its specific interaction using ONE‑GO biosensor, SPR, PRESTO-Tango, and in-cell ELISA assays. In addition, both NTSR1 knockdown and liensinine treatment significantly inhibited cell proliferation/migration and promoting apoptosis. Uncover the potential molecular mechanisms underlying NTSR1‑mediated malignancy using RNA‑seq combined with GO and KEGG enrichment analyses. By systematically investigating the oncogenic potential and therapeutic value of NTSR1 in LUAD, this work not only corroborates NTSR1 as a biomarker for poor prognosis from a multi‑omics perspective but, more importantly, identifies and validates the anti‑tumor efficacy of a novel NTSR1 antagonist. The preliminary insights into its mechanism of action provide a rationale for further development of NTSR1‑targeted therapies.

## Methods and materials

### Bioinformatic analysis of NTSR1 gene expression, function and prognosis

We performed a systematic pan-cancer analysis of NTSR1 expression across 15 cancer types using the online platform Bioinformatics.com.cn. The differential expression of NTSR1 between normal and tumor tissues, as well as its association with clinical stages and tumor purity in LUAD, was further validated through the UALCAN portal (https://ualcan.path.uab.edu/). To investigate the function of NTSR1 in LUAD, we analyzed the genetic alterations of NTSR1 in patient cohorts using the cBioPortal database (http://www.cbioportal.org). Furthermore, the prognostic value of NTSR1 expression was evaluated by overall survival analysis via the Kaplan–Meier Plotter tool (https://www.kmplot.com/analysis/). Finally, we explored the relationship between NTSR1 expression and tumor-infiltrating immune cells using the TIMER database (https://cistrome.shinyapps.io/timer/).

### G protein activation assay by ONE-GO biosensor

HEK-293 T cells were seeded at a density of 30,000 cells per well in 96-well white plates (for BRET measurements) and clear plates (for confluence monitoring). At approximately 80% confluence (~ 24 h post-seeding), cells were transfected with pCDH-NTSR1 × Flag-GFP-Puro and ONE-GO plasmids at a 3:1 receptor-to-G protein plasmid ratio using PEI. After 24 h, the medium was aspirated, and cells were washed once with PBS, followed by incubation with 190 μL of BRET buffer at 37 °C for 30 min. Under light-protected conditions, Nano-Glo® Luciferase Assay substrate (Promega, #N1120) was added, and after a 5-min equilibration at room temperature, BRET signals were quantified using a microplate reader with an integration time of 0.24 s. Readings were taken every 10 s. The first 10 cycles were recorded as background signal, followed by 60 cycles after drug stimulation. For antagonist assays, the first 10 cycles served as background, after which the agonist NTS was added and 3 min were recorded, followed by another 3 min after the addition of liensinine. The BRET ratio was calculated as the emission ratio at 535 nm (acceptor) to 470 nm (donor).

### β-arrestin recruitment assay by PRESTO-Tango

HEK-293 T cells were plated in 6-cm dishes and transfected at ~ 80% confluence with NTSR1-Tango, β-arrestin2-TEV, and TRE-Tight-Luc plasmids at a 2:1:1 DNA mass ratio using a lipid-based transfection reagent according to the manufacturer’s instructions. 24 h after transfection, cells were trypsinized and seeded into white opaque 96-well plates at 30,000 cells/well and incubated overnight at 37 °C and 5% CO_2_. The following day, complete medium was replaced with 100 μL/well basal medium. Test compounds were prepared as 10 × master mixes in basal medium and added to wells to achieve the indicated final concentrations (vehicle and positive controls included). After an 18-h incubation at 37 °C, 50 μL/well of diluted Bright-Glo Luciferase Assay reagent (Promega, E2610) was added and luminescence was measured on a plate luminometer immediately.

### In-cell ELISA for detection of FLAG-NTSR1

HEK-293 T cells were plated at a density of 30,000 cells per well in 96-well plates. Approximately 24 h after seeding, when cells reached ~ 80% confluence, cells were transfected with pcDNA3.1(+)−3 × Flag NTSR1 using PEI (100 ng DNA per well). 24 h post-transfection, medium was aspirated and cells were fixed with 125 μL/well fixative for 10 min. The fixative was removed and wells were washed twice with PBS. Wells were then washed twice with 100 μL/well blocking buffer (1% FBS in PBS), followed by incubation with 125 μL/well blocking buffer at room temperature for 30 min. Blocking buffer was removed and anti‑FLAG primary antibody was added (1:30,000; 75 μL/well) and incubated at room temperature for 60 min. The primary antibody was removed and wells were washed three times with blocking buffer (100 μL each wash), followed by three washes with PBS. HRP‑conjugated secondary antibody (80 μL/well) was added and incubated according to the manufacturer’s instructions. Chemiluminescent substrate was then applied and chemiluminescence was recorded using a plate reader or luminometer. After two PBS washes, 100 μL/well of crystal violet solution (0.1% w/v) was added and incubated at room temperature for 15 min. The stain was discarded and wells were washed three times with distilled water until background was clear. To solubilize the crystal violet, 100 μL/well 10% acetic acid was added and plates were placed on a shaker for 15 min; absorbance was then read at 600 nm (OD600) on a plate reader. Data were normalized as (chemiluminescence_signal − blank)/(OD600_sample − OD600_blank). All treatments were performed at least in triplicate.

### Surface plasmon resonance (SPR) spectroscopy

SPR experiments were conducted at 25 °C on a Biacore 8 K instrument (Cytiva). A Series S CM5 sensor chip was functionalized using the standard amine-coupling procedure. Briefly, the chip surface was activated with a 1:1 mixture of EDC/NHS for 420 s. FLAG-NTSR1, diluted to 30 µg/mL in 10 mM sodium acetate (pH 4.0), was then immobilized on the experimental flow cell to a final response level of approximately 9850 RU. Remaining active groups were blocked with ethanolamine. A reference flow cell was activated and blocked under identical conditions but without protein. All binding assays were performed using 1 × PBST (PBS containing 0.05% Tween-20) supplemented with 5% (v/v) DMSO as the running buffer. Systematic bulk solvent effects were corrected using a calibration curve generated from buffers containing 4.5% and 5.8% DMSO. For analyte binding, a stock solution of liensinine was first diluted 20-fold in 1.05 × PBST, and then serially diluted two-fold in running buffer to generate a 7-point concentration series, with the highest concentration at 781.25 nM. Each concentration was injected over both flow cells at a constant flow rate of 30 µL/min, with an association phase of 60 s followed by a 60 s dissociation phase. When necessary, the chip surface was regenerated with 10 mM glycine-HCl (pH 2.5). The obtained sensorgrams were double-referenced and solvent-corrected. Binding kinetics and affinity were determined by globally fitting the data to a 1:1 Langmuir binding model using the Biacore Insight Evaluation Software.

### Lentiviral particle production and stable cell line generation

Lentiviral particles were produced in HEK-293 T cells using a three-plasmid system. Briefly, the cells were co-transfected with 5 μg of pLKO.1-NTSR1/pCDH-NTSR1 × Flag-GFP-Puro, 3.75 μg of the packaging plasmid (psPAX2), and 1.25 μg of the envelope plasmid (pMD2.G) using Lipofectamine 3000. Viral supernatants were collected at 48 and 72 h post-transfection, filtered through a 0.45 μm membrane, and stored at –80 °C for future use. For transduction, HCI-H1299、HCI-H1975、PC-9 and A549 cells were cultured in 6-well plates until they reached 70% confluence. The cells were then incubated with 1 mL of viral supernatant supplemented with 8 μg/mL polybrene for 24 h. At 72 h post-transduction, stable polyclonal populations were selected by treating the cells with 2 μg/mL puromycin for 7 days.

### Western blot

Protein extraction was conducted with RIPA lysis buffer supplemented with protease and phosphatase inhibitors. Total protein concentration was measured using a bicinchoninic acid (BCA) assay. Proteins (20 μg per lane) were separated by electrophoresis on 4–12% gradient SDS–polyacrylamide gels at 100 V for 90 min and then transferred onto PVDF membranes at 100 V for 60 min. The membranes were blocked with 5% skim milk in TBST for 1 h at room temperature, followed by incubation with primary antibodies overnight at 4 °C with gentle agitation. After three 5-min washes with TBST, the membranes were incubated with HRP-conjugated secondary antibodies for 1 h at room temperature. Protein bands were detected using ECL Prime substrate and visualized with a ChemiDoc MP Imaging System (Bio-Rad).

### Cell viability assay

Cell viability was determined using the Cell Counting Kit-8 (CCK-8; TargetMol®). Exponentially growing cells were seeded into 96-well flat-bottom plates at a density of 2 × 10^3^ cells per well. After 24 h, the medium was replaced with fresh medium containing the indicated concentrations of compounds. At the designated time points (0, 24, 48, 72, and 96 h), 10 μL of CCK‑8 reagent was added to each well and incubated at 37 °C for 1–2 h. Absorbance was measured at 450 nm using a microplate reader. Each experiment included three independent biological replicates.

### Apoptosis assay

Apoptosis was assessed with Annexin V-APC/DAPI and Annexin V-APC/7-AAD apoptosis detection kits (Elabscience). Cells were washed with PBS and resuspended in 125 μL of 1 × Annexin V binding buffer. Subsequently, 1.25 μL of Annexin V-APC was added, followed by either 1.25 μL of 7-AAD (100 μg/mL) or 1.25 μL of DAPI. After gentle vortexing, samples were incubated in the dark at room temperature for 15 min according to the manufacturer’s protocol. The percentages of Annexin V^+^/DAPI^+^ or Annexin V^+^/7-AAD^+^ cells, representing late‑stage apoptotic populations, were analyzed on a NovoCyte Advanteon VBR flow cytometer and quantified using NovoExpress software (v1.4.1). Each condition was tested in three independent biological replicates (n = 3).

### Reverse transcription and quantitative real-time PCR

Total RNA was extracted from cultured cells with TRIzol reagent (Vazyme). First-strand cDNA was synthesized from 500 ng of total RNA using the PrimeScript™ FAST RT Reagent Kit with gDNA Eraser (Takara Bio, Dalian, China). Quantitative real-time PCR (qPCR) was performed using TB Green™ Premix Ex Taq™ II (Tli RNaseH Plus) on a QuantStudio 5 Pro Real-Time PCR System (Applied Biosystems). Gene-specific primer sequences are provided in Table 1. GAPDH served as the internal reference control, and relative gene expression levels were calculated using the 2^−ΔΔCt^ method.

**Table 1:** The primers for RT-qPCR analysis of DEGs

Gene	Forward (5′−3′)	Reverse (5′−3′)
GAPDH	GGAGCGAGATCCCTCCAAAAT	GGCTGTTGTCATACTTCTCATGG
PTGS2	GGTTGCTGGTGGTAGGAATGTTC	CTGGTATTTCATCTGCCTGCTCTG
SPR1	ATTGGGACAGCCGTGGGAAG	ATCGGAATGCTCATTGCTCTCATC
ABCG1	TGAGACGGACCTGCTGAATGG	CCGAGGCAAGGAGGAGAAGC
ABCA1	CCTGAAGCCAATCCTGAGAACAC	ACCTCCTGTCGCATGTCACTC
CASP4	TGGAGGGAATCTGCGGAACTG	GCCTGGACAATGATGACCTTGG

### Transcriptome sequencing

Transcriptome sequencing (RNA‑seq) was performed on cells treated with vehicle control or experimental compounds at the indicated time points. Library preparation and high‑throughput sequencing were carried out by BENAGEN Technology. Raw sequencing reads were assessed for quality using FastQC, followed by adapter trimming and removal of low‑quality bases (Phred score < 30) with Trimmomatic. Cleaned reads were aligned to the human reference genome (GRCh38/hg38) using STAR aligner (v2.7.10a). Differential gene expression analysis was performed using DESeq2, with significant changes defined as |log_2_(fold change)|> 1 and adjusted P < 0.05. Functional enrichment analysis of Gene Ontology (GO) terms and KEGG pathways was conducted with the ClusterProfiler package.

## Results

### Bioinformatics analysis of NTSR1 gene expression, function and prognosis

**Fig. 1:**
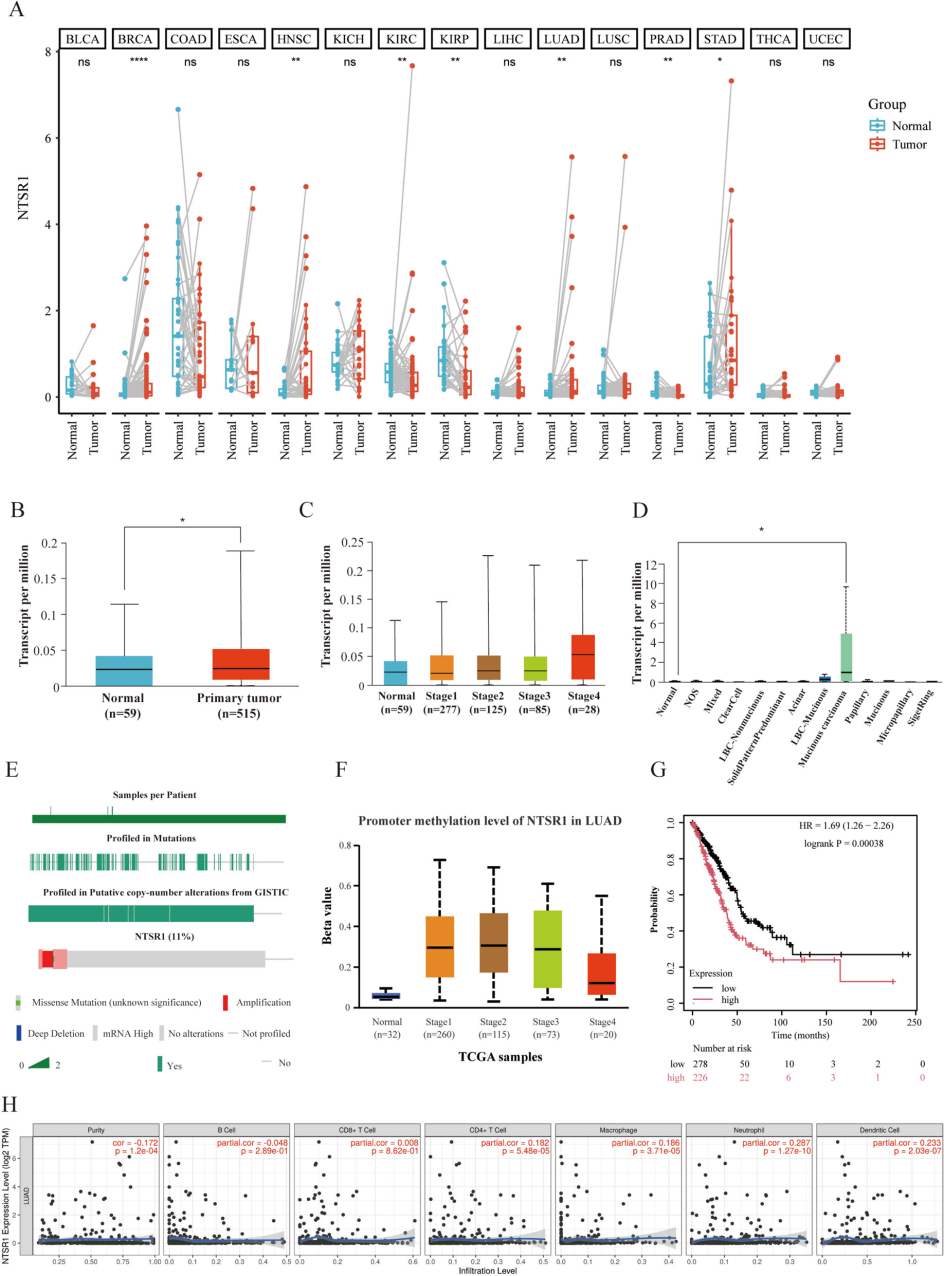
Bioinformatics analysis of NTSR1 gene expression, function and prognosis. **A** Pan-cancer analysis of NTSR1 expression. The mRNA expression levels of NTSR1 across 15 cancer types compared with their corresponding normal tissues (data sourced from the Bioinformatics.com.cn platform). Statistical significance was determined as follows: ns, not significant (p ≥ 0.05); *p < 0.05; **p < 0.01; ****p < 0.0001. **B** Differential expression of NTSR1 in LUAD. Comparison of NTSR1 expression between normal and LUAD tissues. *p < 0.05. **C** NTSR1 expression across pathological stages in LUAD. The association between NTSR1 mRNA levels and different tumor stages (I-IV) in LUAD. **D** NTSR1 expression in LUAD histological subtypes. Analysis of NTSR1 expression levels across different histological subtypes of LUAD. **E** Genetic variation spectrum analysis of NTSR1. **F** Analysis of methylation levels in the NTSR1 promoter region of LUAD tissues and normal lung tissues. **G** Prognostic value of NTSR1 in LUAD. Kaplan–Meier survival curves depicting the correlation between NTSR1 expression and overall survival (OS) in LUAD patients. High and low expression groups are shown in red and blue, respectively. Log-rank p < 0.05 was considered statistically significant. **H** Correlation between NTSR1 expression and immune cell infiltration. Scatter plots showing the relationship between NTSR1 expression levels and the abundance of specific tumor-infiltrating immune cells in LUAD. The blue solid line represents the trend fitted by locally estimated scatterplot smoothing (LOESS), with the gray shaded area indicating the 95% confidence interval

Our systematic pan-cancer analysis reveals that the expression of NTSR1 is significantly dysregulated across multiple tumor types, suggesting its potential role as a widespread cancer-associated gene (Fig. 1A). Specifically, the consistent upregulation of NTSR1 in Breast cancer (BRCA), Head and Neck Squamous Cell Carcinoma (HNSC), Kidney renal clear cell carcinoma (KIRC), LUAD, and Stomach Adenocarcinoma (STAD) strongly implies its likely oncogenic function in these cancers, possibly mediated through common pro-tumorigenic mechanisms. Conversely, its downregulation in Kidney papillary cell carcinoma (KIRP) and Prostate adenocarcinoma (PRAD) indicates that the function of NTSR1 may be highly context-dependent, potentially involving distinct regulatory networks in different cellular environments. Focusing on LUAD, the overexpression of NTSR1 was further validated (Fig. 1B), reinforcing the key finding of its aberrant activation in this cancer. More importantly, NTSR1 expression showed a positive correlation with tumor stage (Fig. 1C), suggesting that its upregulation may not only be an early event in lung carcinogenesis but also closely associated with disease progression and increased aggressiveness. Thus, its expression level could serve as a potential molecular marker for evaluating disease stage. Furthermore, among the histological subtypes of LUAD, the mucinous subtype exhibited the most pronounced NTSR1 overexpression (Fig. 1D). This specific association strongly indicates that aberrant NTSR1 expression may be linked to the development of particular pathological subtypes, especially the mucinous variant.

Based on these findings, we further analyzed the genetic alteration profile of NTSR1 (Fig. 1E). The results showed that approximately 11% of tumor samples harbored genetic alterations in NTSR1, with missense mutations being the most common (6%), followed by gene amplification (3%) and deep deletion (2%). Notably, 4.3% of cases exhibited significant mRNA overexpression without detectable DNA-level alterations, suggesting possible epigenetic regulation. To test this hypothesis, we compared the methylation levels in the NTSR1 promoter region between LUAD tissues and normal lung tissues (Fig. 1F). In contrast to the typical silencing pattern observed for tumor suppressor genes, the promoter methylation of NTSR1 was significantly higher in tumor tissues than in normal tissues, indicating that its overexpression is not driven by promoter demethylation. This inverse regulatory pattern implies that NTSR1 expression may be governed by other epigenetic mechanisms. Its high expression in LUAD is thus likely a complex process driven by a combination of genetic and epigenetic events.

To evaluate the clinical relevance of these molecular features, we further assessed the prognostic value of NTSR1 expression. Kaplan–Meier survival analysis revealed that high NTSR1 expression was significantly associated with shorter overall survival (OS) in LUAD patients, suggesting its potential as an independent prognostic factor for poor outcome (Fig. 1G). Given the critical role of the tumor immune microenvironment in cancer progression and treatment response, we subsequently examined the correlation between NTSR1 expression and immune cell infiltration. Our analysis showed that NTSR1 expression levels were positively correlated with the infiltration of CD8⁺ T cells, CD4⁺ T cells, macrophages, neutrophils, and dendritic cells, whereas a negative correlation was observed with B cell abundance and tumor purity (Fig. 1H). This complex immune association pattern suggests that NTSR1 may contribute to shaping an immunosuppressive microenvironment in LUAD by modulating the recruitment or function of specific immune cells.

### Screening and characterization of NTSR1 antagonists

As described in the introduction, the binding of NTS to NTSR1 primarily drives tumor cell proliferation, survival, migration, and metastasis via the classical “Gq–Ca^2^⁺–PKC–MAPK” signaling axis. Therefore, screening compounds that antagonize NTSR1 and inhibit Gq signaling is theoretically a promising strategy to suppress tumor progression. In our previous large-scale screening efforts, we identified liensinine as a potential interactor of NTSR1 (unpublished data), however, the biological function of this interaction and its downstream signaling mechanisms remain unclear.

**Fig. 2:**
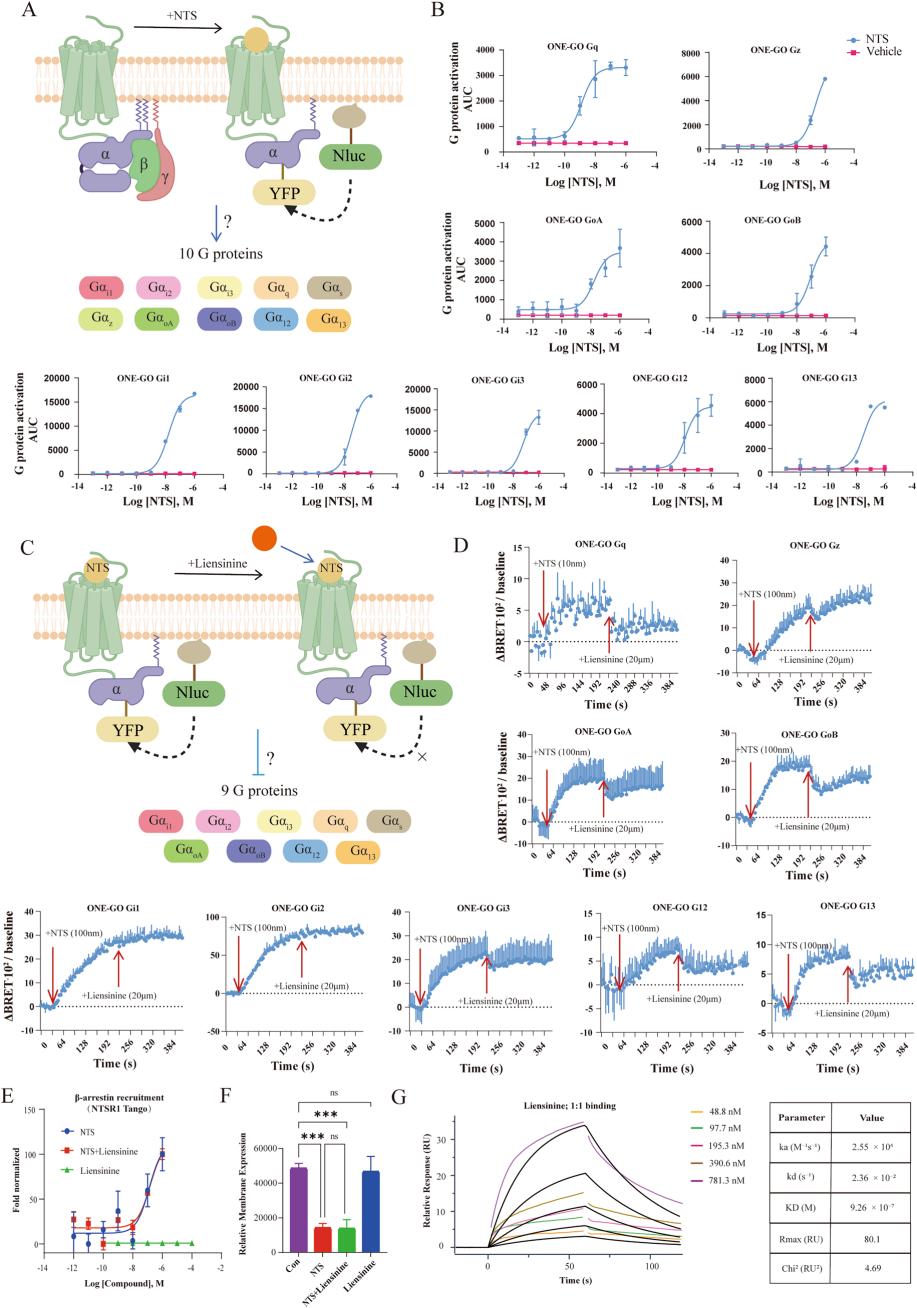
Liensinine interacts with NTSR1 and inhibits its downstream G protein signaling pathways. **A** Schematic diagram of ONE-GO biosensor detection system. **B** Signal profiles of multiple G protein subtypes upon NTS-induced NTSR1 activation. **C** Schematic diagram illustrating the broad-spectrum inhibitory effects of liensinine on NTSR1‑mediated activation of nine Gα subunits as detected by the ONE‑GO biosensor. **D** Liensinine inhibits NTS-induced activation of pathways. **E** PRESTO-Tango reveals that liensinine does not inhibit NTS-induced β-arrestin recruitment to NTSR1. **F** Measurement of receptor internalization (In-cell ELISA) indicates that liensinine does not prevent NTS-mediated reduction of NTSR1 membrane expression. Statistical significance was assessed by one‑way analysis of variance (one‑way ANOVA) followed by Dunnett’s multiple comparisons test. Asterisks in figures indicate statistical significance as follows: ns (not significant); *P < 0.05; **P < 0.01; ***P < 0.001; ****P < 0.0001. **G** Direct binding analysis by SPR demonstrates that liensinine interacts with NTSR1

To elucidate the specific signaling mechanism, we first characterized the downstream signaling profile of NTS-induced NTSR1 activation using the ONE-GO biosensor system (Fig. 2A). The results revealed that NTS activation of NTSR1 significantly stimulated multiple G protein subtypes, including Gi/o, Gq/11, and G12/13, but did not activate the Gs pathway. Notably, NTSR1 exhibited the strongest coupling efficacy to the canonical oncogenic Gq subtype, which was markedly more potent than its coupling to the Gi/o family or G12/13 subtypes (Fig. 2B). Subsequently, to evaluate the inhibitory effect of liensinine on NTS-induced NTSR1 signal transduction, we treated cells with 20 µM liensinine following NTS stimulation and monitored its impact on downstream G protein coupling (Fig. 2C). The results demonstrated that liensinine potently and specifically blocked the NTSR1-mediated classical Gq signaling axis. Concurrently, liensinine exhibited broad-spectrum inhibitory activity against Go, G12/13, and Gi3 subtypes, although its effects on these pathways were less complete compared to its nearly complete suppression of Gq signaling. Interestingly, we found that liensinine had minimal inhibitory effect on the coupling of Gz, Gi1, and Gi2, with particularly negligible suppression observed for Gi1 and Gi2 (Fig. 2D).

Building upon these findings, we further examined β-arrestin recruitment using the PRESTO-Tango assay. The results showed that NTS stimulation alone effectively recruited β-arrestin in a concentration-dependent manner. When cells were co-treated with NTS and liensinine, β-arrestin recruitment remained concentration-dependent and was not markedly blocked. Notably, liensinine alone did not induce significant β-arrestin recruitment (Fig. 2E). To investigate the effect of liensinine on receptor membrane localization, we assessed the membrane expression of FLAG-NTSR1 via in-cell ELISA. Treatment with NTS significantly reduced the membrane expression of NTSR1, indicating receptor internalization. This internalization effect persisted when NTS was combined with liensinine. In contrast, liensinine alone did not cause a significant change in NTSR1 membrane expression (Fig. 2F).

To determine whether liensinine exerts its effect through direct binding to NTSR1, we performed SPR spectroscopy. Purified human NTSR1 was immobilized on a CM5 sensor chip. Injecting increasing concentrations of liensinine resulted in concentration-dependent binding responses (Fig. 2G, left panel). The sensoryrams were fitted using a 1:1 Langmuir binding model, which yielded an equilibrium dissociation constant (K_D) of 9.26 × 10⁻⁷ M (Fig. 2G, right panel). No significant binding was detected to a mock-immobilized reference surface, confirming the specificity of the interaction. These data establish liensinine as a direct binder of NTSR1 with micromolar affinity.

### Liensinine inhibits lung cancer cell proliferation, migration, and invasion by regulating NTSR1

**Fig. 3:**
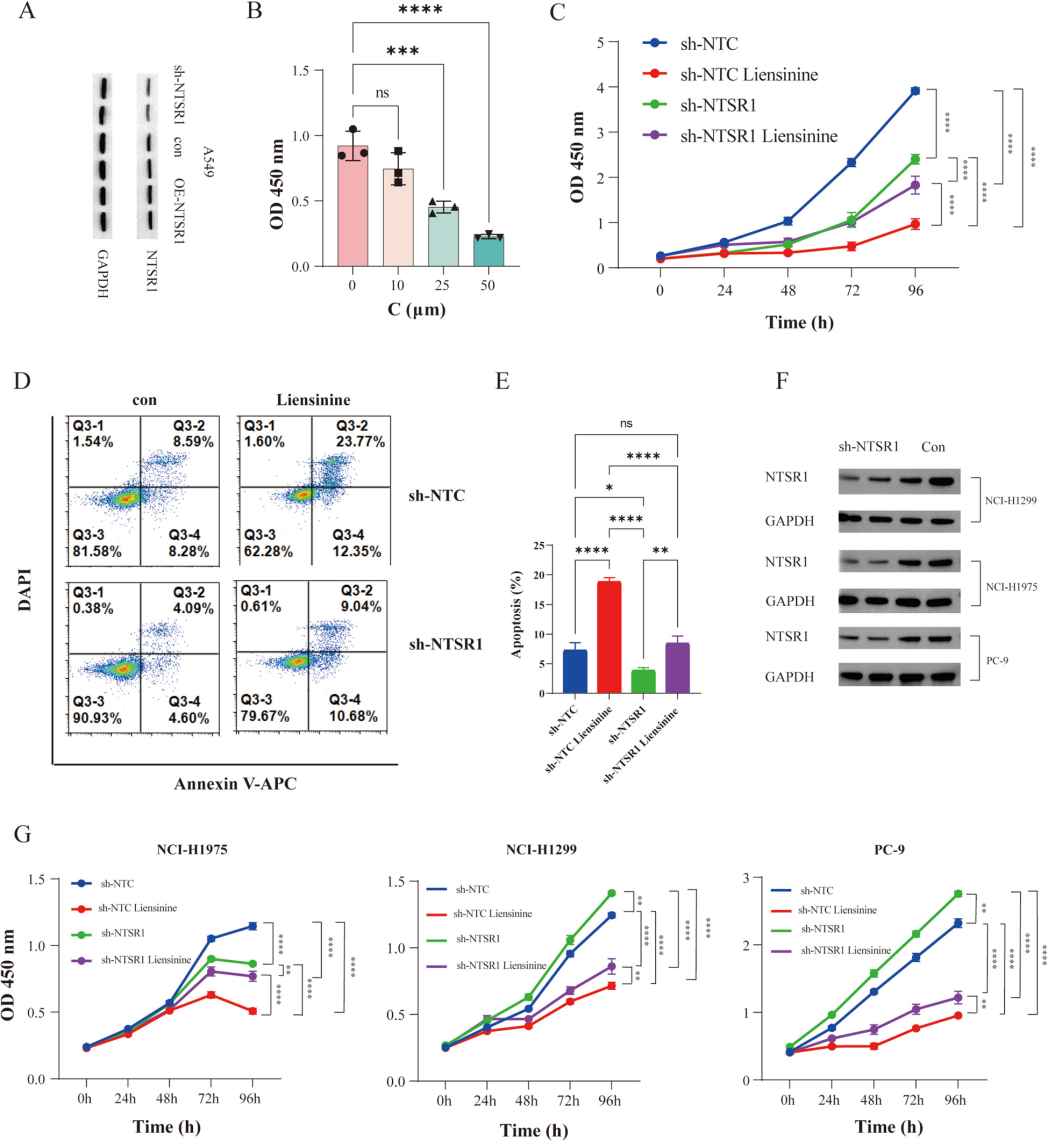
The inhibitory effect of liensinine on cell proliferation and its promotion of apoptosis are dependent on NTSR1. Statistical significance was assessed by one‑way analysis of variance (one‑way ANOVA) followed by Dunnett’s multiple comparisons test. Asterisks in figures indicate statistical significance as follows: ns (not significant); *P < 0.05; **P < 0.01; ***P < 0.001; ****P < 0.0001. **A** Western blot analysis of sh-NTSR1 and OE-NTSR1 efficiency expression in A549 cells. **B** A549 cells were cultured in liensinine at concentrations ranging from 0 to 50 μM for 48 h, and cell viability was assessed using the CCK-8 assay. **C** A549 cells were cultured in liensinine at specified concentrations for 24–96 h to assess cell viability. **D** and **E** Percentage of apoptotic cells after liensinine treatment, determined by fluorescence-enhanced flow cytometry. **F** Western blot analysis of sh-NTSR1 efficiency expression in HCI-H1299, HCI-H1975 and PC-9 cells. **G** HCI-H1299, HCI-H1975 and PC-9 cells were cultured in liensinine at specified concentrations for 24–96 h to assess cell viability. Cell viability (CCK8)

To evaluate the anti-LUAD potential of liensinine, we established non-targeting control (sh-NTC) and stable NTSR1 knockdown (sh-NTSR1) models in A549 cell lines, and efficient knockdown of NTSR1 was confirmed by western blot analysis (Fig. 3A). We assessed the effect of liensinine on A549 cell viability using CCK-8 assays. The results showed that liensinine significantly inhibited A549 cell proliferation in a concentration-dependent manner, with an IC_50_ value of 20 μM (Fig. 3B). Based on this finding, we selected 20 μM as the working concentration of liensinine for subsequent experiments. Further experiments revealed that either NTSR1 knockdown or liensinine treatment alone effectively reduced the viability of A549 cells (Fig. 3C). Notably, liensinine treatment alone exhibited a more significant anti-proliferative effect compared to its application in the context of NTSR1 knockdown, indicating that NTSR1 is required for the tumor-suppressive function of liensinine. In addition, Annexin V-APC/DAPI double-staining flow cytometry analysis demonstrated that both NTSR1 knockdown and liensinine treatment independently induced significant apoptosis in A549 cells. Importantly, liensinine treatment alone induced a more substantial apoptotic effect compared to its use under NTSR1 knockdown conditions (Fig. 3D, E), further confirming that the pro-apoptotic effect of liensinine depends on the presence of NTSR1. To assess the generalizability of our findings beyond A549 cells, we evaluated the effect of liensinine on the viability of three additional LUAD cell lines (HCI-H1299、HCI-H1975 and PC-9). Consistent with the results in A549 cells, liensinine treatment alone exhibited a more significant anti-proliferative effect compared to its application in the context of NTSR1 knockdown (Fig. 3F, G).

### Identification of differentially expressed genes in response to OE-NTSR1 and liensinine

**Fig. 4:**
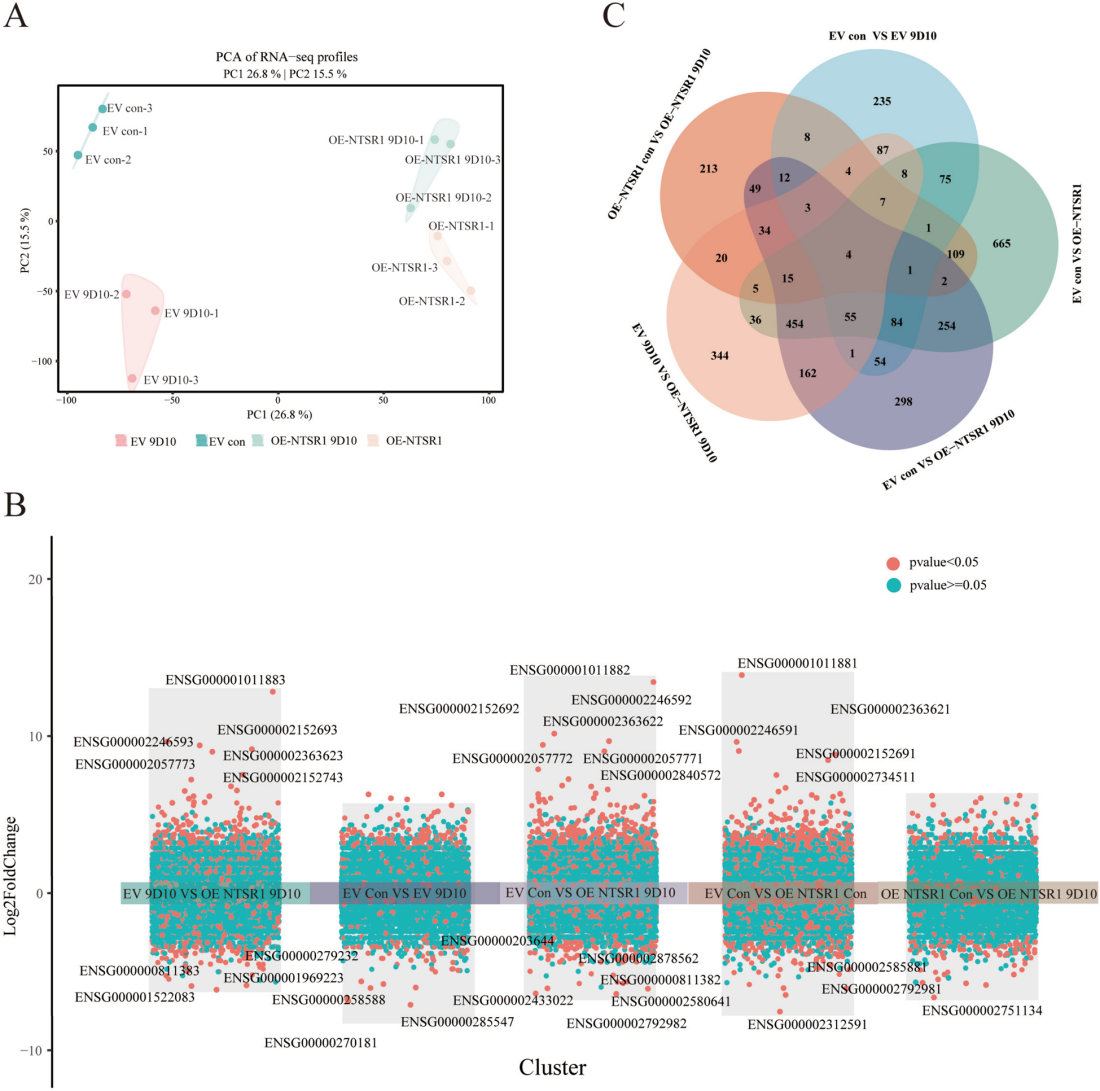
Transcriptomic profiling reveals molecular characteristics associated with NTSR1 overexpression and liensinine synergy. **A** PCA depicting the overall distribution of gene expression profiles in A549 cells across different treatment conditions. Each data point represents an individual sample, with colors indicating experimental groups. **B** Heatmap of hierarchically clustered differentially expressed genes. **C** Venn diagram illustrating the overlap of differentially expressed genes identified across the four experimental comparisons

The malignant progression of LUAD is driven by multiple constitutively activated oncogenic signaling pathways. To elucidate the molecular pathways regulated by the liensininel (9D10)–NTSR1 axis during LUAD progression, we performed deep RNA sequencing under four isogenic conditions: EV, EV + liensinine, OE-NTSR1, and OE-NTSR1 + liensinine, with three biological replicates per group. Principal component analysis (PCA) based on gene expression levels (TPM) revealed four distinct clusters, closely reflecting the experimental design and indicating high intra-group consistency and minimal batch effects (Fig. 4A). PC1, accounting for 26.8% of the total variance, clearly separated NTSR1-overexpressing clones from EV along a single trajectory, identifying NTSR1 as the primary driver of transcriptional differences. PC2 (15.5% of variance) stably distinguished liensinine-treated from vehicle-treated samples in the NTSR1-high background, suggesting that liensinine exerts a consistent, though modest, effect on gene expression.

Differential expression analysis (|log_2_FC|≥ 1, p < 0.05) was visualized using volcano plots (Fig. 4B). Compared to the EV con, liensinine monotherapy (EV liensinine) significantly altered 640 genes (449 up, 191 down). NTSR1 overexpression alone (OE-NTSR1 Con) induced more substantial changes, modulating 1776 genes (1023 up, 753 down) compared to the control. Strikingly, in the NTSR1-overexpressing background, liensinine treatment (OE-NTSR1 liensinine) resulted in a more focused set of 488 altered genes (258 up, 230 down) compared to its respective control. Furthermore, direct comparison between EV liensinine and OE-NTSR1 liensinine revealed 1540 differentially expressed genes (987 up, 553 down), highlighting the profound impact of NTSR1 status on the cellular response to liensinine. Most importantly, the combined effect of NTSR1 overexpression and liensinine (OE-NTSR1 liensinine) versus the baseline control (EV Con) identified 1483 differentially regulated genes (927 up, 556 down), underscoring a potent synergistic transcriptional reprogramming.

Following the identification of differentially expressed genes (DEGs) across all comparisons, a Venn diagram was used to visualize the overlap and specificity of transcriptional changes under the four treatment conditions (Fig. 4C). The analysis revealed a core set of four genes common to all comparisons. These commonly regulated genes likely represent a fundamental transcriptional network essential for the synergy between NTSR1 overexpression and liensinine, potentially acting as key mediators of the combined effect.

### Functional enrichment analysis of differentially expressed genes

**Fig. 5:**
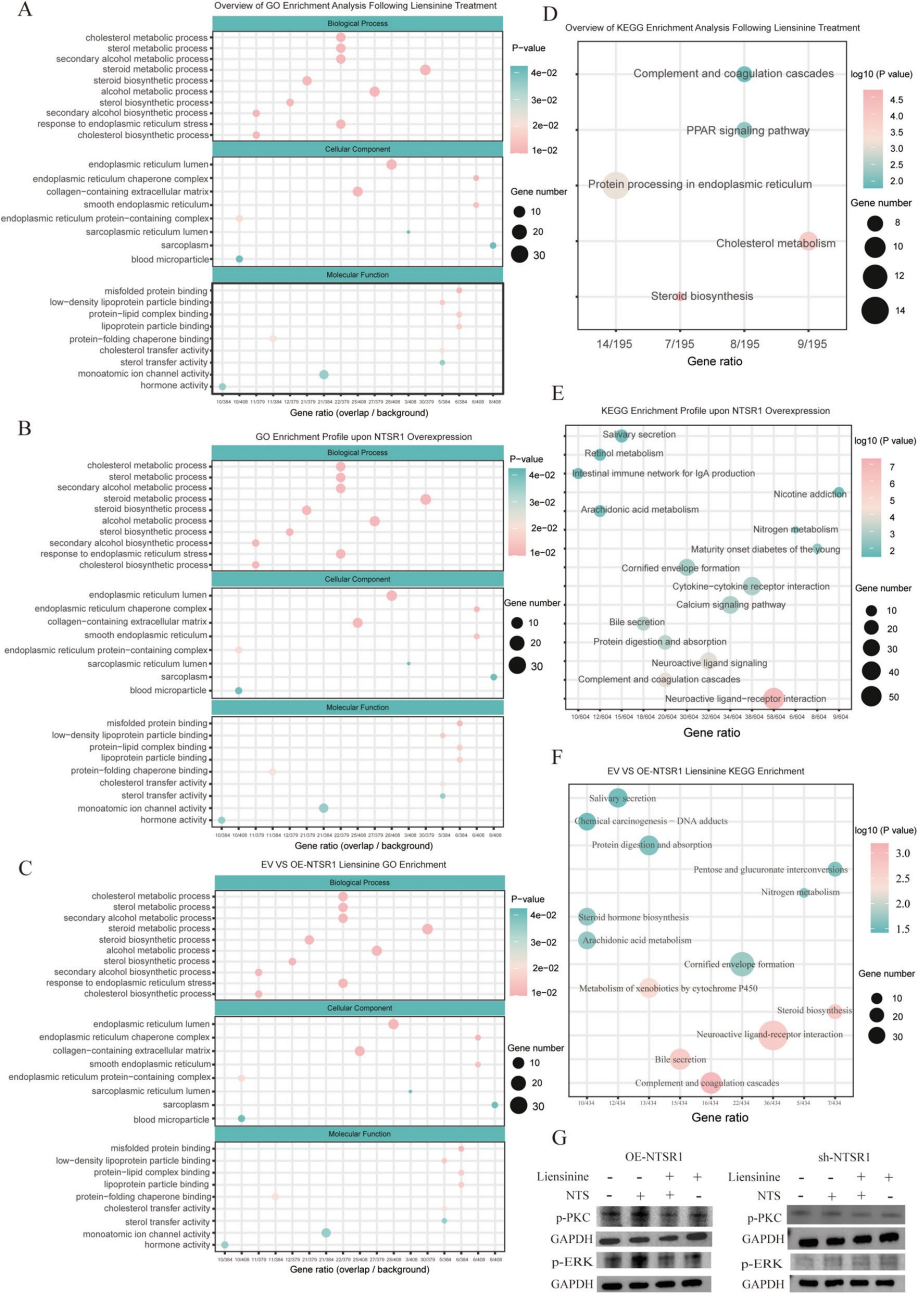
Transcriptional reprogramming by NTSR1 overexpression and liensinine, and validation of downstream signaling dependency

To further elucidate the biological roles of NTSR1 overexpression and its potential antagonist, liensinine, this study categorized five experimental groups (EV vs. EV + liensinine, EV vs. OE-NTSR1, EV vs. OE-NTSR1 + liensinine, OE-NTSR1 vs. OE-NTSR1 + liensinine, EV liensinine vs. OE-NTSR1 liensinine) into three distinct treatment conditions: NTSR1 overexpression, liensinine drug treatment, and the combination of overexpression with drug treatment. Functional enrichment analysis of differentially expressed genes under these conditions revealed that Gene Ontology (GO) analysis did not indicate broad functional alterations (Fig. 5A, B, C). In contrast, KEGG pathway enrichment uncovered a series of highly specific signaling pathways closely associated with tumor biology, thereby providing a solid molecular basis for our phenotypic hypothesis (Fig. 5D, E, F).

In the NTSR1 overexpression group, we observed significant activation of multiple pathways directly linked to tumorigenesis and progression (Fig. 5D). The enrichment of the neuroactive ligand-receptor interaction and calcium signaling pathways is highly consistent with the identity of NTSR1 as a GPCR, suggesting that its overexpression may sustain the activation of downstream pro-proliferative and survival signals through autocrine or paracrine mechanisms. Furthermore, the activation of the cytokine-cytokine receptor interaction pathway indicates a potential remodeling of the immune response within the tumor microenvironment, which is often associated with tumor immune evasion. Notably, perturbations in metabolic pathways such as arachidonic acid metabolism and bile secretion further revealed a potential novel function of NTSR1 in reprogramming cellular metabolism to support rapid growth. Together, these findings outline a molecular map through which NTSR1 may exert its pro-tumorigenic effects by coordinately driving pro-proliferative signaling, an inflammatory microenvironment, and metabolic adaptation.

In the group treated with liensinine alone, the enriched pathways displayed a pattern distinct from that of the overexpression group (Fig. 5E). Drug treatment specifically led to enrichment in sterol biosynthesis, cholesterol metabolism, and protein processing in the endoplasmic reticulum. These alterations typically point to two key anti-cancer mechanisms: First, the inhibition of cholesterol and its derivatives (such as certain hormones) can directly restrict the biomembranes and signaling molecules required for tumor growth. Second, the induction of endoplasmic reticulum stress may trigger the unfolded protein response, thereby suppressing protein synthesis and potentially inducing apoptosis. Concurrently, the enrichment of the PPAR signaling pathway further corroborates the profound metabolic reprogramming elicited by the drug.

The most critical evidence emerged from the “overexpression + liensinine” combination treatment group (Fig. 5F). In this group, the enrichment levels of many pro-tumorigenic signaling pathways characteristic of NTSR1 overexpression—most notably neuroactive ligand-receptor interactions—were markedly attenuated. The calcium signaling pathway was no longer enriched, potentially indicating that liensinine blocks NTS-mediated activation of the Gq-coupled signaling pathway downstream of NTSR1. Meanwhile, pathways representing the effects of liensinine (e.g., sterol biosynthesis) and those related to both conditions (e.g., complement and coagulation cascades, bile secretion) were enriched. This shift in the transcriptional profile—from “pro-signaling transduction” towards “pro-metabolic regulation”—strongly demonstrates that liensinine effectively counteracts the oncogenic activity of NTSR1 and resets the cellular transcriptional program from a proliferation-driven state to a drug-controlled state characterized by enhanced metabolic stress.

To further validate the effects of NTSR1 overexpression and liensinine treatment on downstream signaling at the protein level, we performed Western blot analysis. The results showed that in OE‑NTSR1 cells, stimulation with NTS significantly enhanced the phosphorylation levels of PKC and ERK compared with the control group, indicating that NTSR1 activation effectively promotes the phosphorylation of both PKC and ERK signaling pathways. In contrast, when cells were treated with both NTS and liensinine, the signal intensities of p‑PKC and p‑ERK were markedly reduced relative to the NTS‑alone group (Fig. 5G). Treatment with liensinine alone also decreased the phosphorylation levels of p‑PKC and p‑ERK, suggesting that liensinine itself exerts a certain inhibitory effect on basal signaling activity. Conversely, in sh-NTSR1 cells, none of the three treatments—NTS alone, NTS combined with liensinine, or liensinine alone—produced significant changes in p-PKC or p-ERK levels compared with the control (Fig. 5G). These results further confirm that the inhibitory effect of liensinine on the downstream PKC and ERK signaling pathways is dependent on NTSR1 expression.

### The core gene signature mediating the NTSR1–liensinine interaction

To substantiate the pivotal role of the four core overlapping genes identified from our transcriptomic analysis (Fig. 4C), which are hypothesized to constitute the fundamental transcriptional network underlying the synergistic interplay between NTSR1 overexpression and liensinine treatment, we performed a two-tiered validation strategy. This encompassed experimental confirmation of their expression patterns in vitro and an assessment of their clinical relevance in LUAD patient cohorts.

**Fig. 6:**
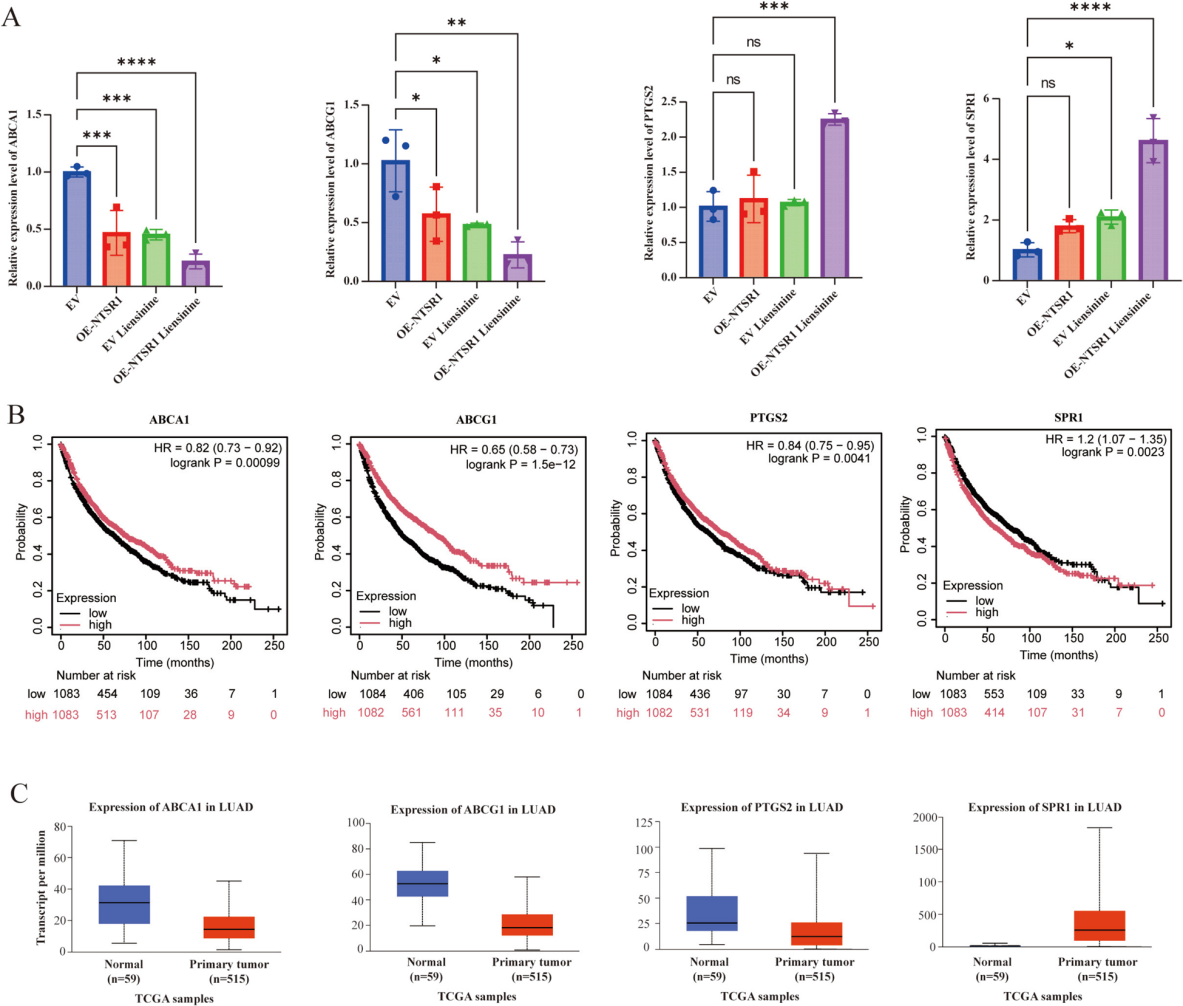
Transcriptome-based validation and public-database mining identify potential clinical targets in LUAD. **A** RT-qPCR verification of the expression changes of the four core genes (*p < 0.05, **p < 0.01, ***P < 0.001, ****P < 0.0001). **B** Kaplan–Meier Plotter survival analysis validating the four core genes. **C** The UALCAN dataset shows the expression of genes in LUAD (red boxplot) and normal (grey boxplot) samples

We sought to validate the expression changes of these four core genes in isogenic cell line models using RT‑qPCR. The qPCR results strongly corroborated our RNA‑seq findings. Specifically, under the four experimental conditions—EV, EV + liensinine, OE-NTSR1, and OE-NTSR1 + liensinine—the expression trends of *PTGS2*, *SPR1*, *ABCG1*, and *ABCA1* closely matched those from the sequencing data (Fig. 6A). This independent verification confirms the reliability of our transcriptomic dataset and reinforces the status of these genes as a consistent molecular signature responsive to both NTSR1‑driven tumorigenesis and liensinine-mediated antagonism. Next, to extend beyond our cellular model and evaluate the translational relevance of this core gene set, we interrogated publicly available LUAD patient data from the UALCAN platform. We analyzed the expression patterns of these four genes in LUAD and their collective impact on patient survival. Kaplan–Meier analysis revealed that high expression of *ABCA1*, *ABCG1*, and *PTGS2* was associated with more favorable survival outcomes, with *ABCG1* showing the strongest protective effect. In contrast, high *SPR1* expression indicated poor prognosis (Fig. 6B). Concurrently, comparison of gene expression between lung cancer tissues and adjacent normal tissues showed that *SPR1* was significantly upregulated in tumors, whereas *ABCA1*, *ABCG1*, and *PTGS2* exhibited higher expression in normal tissues (Fig. 6C). Together, these results suggest that this gene signature may hold value for prognostic assessment and therapeutic targeting in cancers such as LUAD.

## Discussion

This study provided multi-omics evidence supporting the central role of NTSR1 in LUAD. We demonstrated that NTSR1 is significantly overexpressed in LUAD tissues, and its expression level strongly correlates with advanced TNM stage and poor overall survival, aligning with its reported roles in other solid tumors [28–32]. These findings suggest that aberrant NTSR1 activation is a key oncogenic driver across cancer types. Furthermore, our analysis linked NTSR1 overexpression to the remodeling of the tumor immune microenvironment, offering a new perspective for understanding LUAD heterogeneity and treatment resistance. Previous studies demonstrated that NTSR1 may contribute to an immunosuppressive microenvironment via crosstalk with pathways such as PKC/MAPK and EGFR, or by regulating the expression of immune-related genes, potentially diminishing the efficacy of immune checkpoint inhibitors [27, 33–35]. Thus, targeting NTSR1 presents a dual therapeutic strategy, directly inhibit tumor growth and overcoming resistance to existing targeted and immunotherapies.

Although prior studies have suggested a potential role for NTSR1 in lung cancer, we provide direct genetic evidence through loss-of-function experiments, confirming that NTSR1 is indispensable for maintaining the malignant phenotype of LUAD cells. A key breakthrough of this work is the first identification of the natural product liensinine as a potent NTSR1 antagonist. Subsequent in-vitro pharmacodynamic evaluations showed that liensinine phenocopied the effects of NTSR1 knockdown, effectively inhibiting cell proliferation and migration while promoting apoptosis. This discovery provides a novel and promising lead compound for developing NTSR1-targeted therapies, and offers pharmacological validation that NTSR1 is a “druggable” target for LUAD treatment [36].

Canonical NTSR1 signal transduction relies on the Gα_q/11_-PLC-PKC axis [37, 38], a mechanism was validated in our earlier ONE-GO biosensor assays. Our RNA-seq data revealed significant enrichment of the Ca^2^⁺ signaling pathway, accompanied by altered expression of its key genes, thereby providing support for the aforementioned mechanism. In contrast, liensinine treatment abolished the Ca^2^⁺ pathway enrichment and attenuated neuroactive ligand-receptor interactions. Instead, pathways related to metabolism, such as steroid biosynthesis, cholesterol metabolism, and endoplasmic reticulum protein processing were significantly enriched. This transcriptional shift from a “pro-signaling transduction” to a “pro-metabolic regulation” profile indicates that liensinine effectively antagonizes the oncogenic activity of NTSR1. It exerts its anti-tumor effects by redirecting the cellular transcriptional program from a proliferation-driven state to one dominated by metabolic stress responses. These findings are consistent with our data from the ONE-GO biosensor, which showed that liensinine inhibits Gq signaling.

Furthermore, we observed that NTSR1-coupled Gi/o signaling was less suppressed upon drug treatment, suggesting that liensinine may promote sustained coupling of NTSR1 to Gi/o proteins. This could activate the Gi/o → AC inhibition → cAMP decrease → AMPK activation axis, thereby shifting the transcriptional program toward genes involved in “energy metabolism/fatty acid oxidation/glycolysis”. This shift phenotypically presenting as a transcriptional contrast characterized by “metabolic enrichment and loss of Gq signaling”. Therefore, KEGG pathway analysis not only validated the hypothesis that NTSR1 acts as an oncogene by activating multiple signaling networks to drive tumor progression but also delineated the mode of action of its antagonist, liensinine. Specifically, liensinine appears to antagonize pro-tumorigenic signaling by interfering with NTSR1 coupling to Gq/11 proteins, while potentially biasing receptor signaling preference toward the Gi/o-AMPK metabolic regulatory axis. Moreover, the discovery of the core gene signature (*PTGS2*, *SPR1*, *ABCG1*, *ABCA1*) further elucidates the molecular interface between NTSR1 activation and pharmacological inhibition.

Despite our findings, this study has certain limitations. Our conclusions are derived from in vitro cell line models. Although we have validated the anti-LUAD effect of liensinine across multiple cell lines (A549, HCI-H1299、HCI-H1975 and PC-9), the clinical relevance requires further investigation. Future studies should analyze NTSR1 expression in primary LUAD patient samples and correlate it with clinicopathological features and patient outcomes. Furthermore, it should be noted that this study has two main limitations regarding in vivo evaluation. First, the inhibitory effects of liensinine on the NTSR1-Gq signaling pathway and its downstream phenotypes were only validated in vitro using cell models. No in vivo tumor models in mice have been established to directly demonstrate its antitumor efficacy or to determine the exposure–response (PK/PD) relationship [39]. Second, existing literature suggests that liensinine has low oral bioavailability (reported to be low) [40], indicating that if it is to be developed as a systemic therapeutic agent, its pharmacokinetic/pharmacometabolic (ADME) properties and route of administration need to be thoroughly optimized.

Based on these limitations, follow-up studies should include the following: dose–response and tolerability experiments in suitable in vivo tumor models (e.g., xenograft or immunocompetent syngeneic models) to evaluate antitumor activity; systematic ADME/toxicity assessments covering solubility, hepatic microsomal metabolism, permeability, plasma protein binding, CYP enzyme interactions, and hERG inhibition potential.

Nevertheless, the cellular and mechanistic evidence provided in this study establishes liensinine as a potential lead compound targeting the NTSR1-Gq pathway, laying an important foundation for subsequent in vivo studies and pharmacokinetic optimization.

## Conclusion

In conclusion, this study establishes NTSR1 as a critical oncogenic driver and a promising therapeutic vulnerability in LUAD. Through a multi-faceted approach, we have not only corroborated its prognostic significance and role in shaping the tumor immune landscape but also, for the first time, identified the natural compound liensinine as a potent and selective NTSR1 antagonist. Mechanistically, liensinine exerts its anti-tumor effects by preferentially inhibiting the canonical Gq-mediated Ca^2+^-PKC-MAPK signaling axis and shifting the cellular transcriptional program towards metabolic stress responses. Overall, the data encourage additional preclinical studies to assess NTSR1‑targeted strategies in LUAD and to determine the therapeutic potential of liensinine.

## Data Availability

Raw data for RNA-seq have been deposited at https://www.ncbi.nlm.nih.gov/bioproject/PRJNA1372826/. The full screening data will be available upon requests to the corresponding authors.

## Abbreviations

LUAD: Lung adenocarcinoma
NSCLC: Non-small cell lung cancer
NTSR1: Neurotensin receptor 1
NTS: Neurotensin
GPCR: G protein-coupled receptor
SPR: Surface plasmon resonance
ELISA: Enzyme-linked immunosorbent assay
PRESTO-Tango: Parallel receptor evaluation and screening via transcriptional output-Tango
CCK-8: Cell Counting Kit-8
RNA-seq: RNA sequencing
RT-qPCR: Reverse transcription quantitative polymerase chain reaction
GO: Gene Ontology
KEGG: Kyoto Encyclopedia of Genes and Genomes
BRET: Bioluminescence resonance energy transfer
ANOVA: Analysis of variance
PKC: Protein kinase C
ERK: Extracellular signal-regulated kinase
MAPK: Mitogen-activated protein kinase
PLC: Phospholipase C
PIP2: Phosphatidylinositol 4,5-bisphosphate
IP3: Inositol 1,4,5-trisphosphate
DAG: Diacylglycerol
FBS: Fetal bovine serum
PBS: Phosphate-buffered saline
PVDF: Polyvinylidene fluoride
BCA: Bicinchoninic acid
DMSO: Dimethyl sulfoxide
DEG(s): Differentially expressed gene(s)
ECL: Enhanced chemiluminescence
PPAR: Peroxisome proliferator-activated receptor
ADME: Absorption, distribution, metabolism, and excretion
PTGS2: Prostaglandin-endoperoxide synthase 2
SPR1: Small proline-rich protein 1
ABCA1: ATP-binding cassette transporter A1
ABCG1: ATP-binding cassette subfamily G member 1

## References

[1] Chiu CH, et al. Challenges of lung cancer control in Asia. EClinMed. 2024;74:102706. 10.1016/j.eclinm.2024.102706. 10.1016/j.eclinm.2024.102706 PMC11701479 39764183

[2] Siegel RL, et al. Cancer statistics, 2025. CA Cancer J Clin. 2025;75(1):10–45. 10.3322/caac.21871. 39817679 10.3322/caac.21871 PMC11745215

[3] Walsh RJ, et al. Resistance to immune checkpoint inhibitors in non‑small cell lung cancer: biomarkers and therapeutic strategies. Ther Adv Med Oncol. 2020;12:1758835920937902. 10.1177/1758835920937902. 32670423 10.1177/1758835920937902 PMC7339077

[4] Kang K, et al. Addressing clinical needs in NSCLC immunotherapy: mechanisms of resistance and promising combination strategies. Cell Rep Med. 2025;6(9):102315. 10.1016/j.xcrm.2025.102315. 40876451 10.1016/j.xcrm.2025.102315 PMC12490215

[5] Sharma P, et al. Primary, adaptive, and acquired resistance to cancer immunotherapy. Cell. 2017;168(4):707–23. 10.1016/j.cell.2017.01.017. 28187290 10.1016/j.cell.2017.01.017 PMC5391692

[6] Zhang Y, et al. Targeting NAT10 inhibits hepatocarcinogenesis via ac4C‑mediated SMAD3 mRNA stability. Exploration. 2025;5(6):20250075. 10.1002/EXP.20250075. 41476650 10.1002/EXP.20250075 PMC12752639

[7] Yang L, et al. Visualization analysis of research progress and trends in coexistence of lung cancer and pulmonary tuberculosis using bibliometrics. Med Adv. 2024;2:144–64. 10.1002/med4.58.

[8] Reck M, et al. Pembrolizumab versus chemotherapy for PD‑L1‑positive non‑small‑cell lung cancer. N Engl J Med. 2016;375(19):1823–33. 10.1056/NEJMoa1606774. 27718847 10.1056/NEJMoa1606774

[9] Doroshow DB, et al. PD‑L1 as a biomarker of response to immune‑checkpoint inhibitors. Nat Rev Clin Oncol. 2021;18(6):345–62. 10.1038/s41571-021-00473-5. 33580222 10.1038/s41571-021-00473-5

[10] Santhanam B, et al. Exploring GPCR signaling pathway networks as cancer therapeutic targets. Cell Genom. 2024;4(5):100560. 10.1016/j.xgen.2024.100560. 38723606 10.1016/j.xgen.2024.100560 PMC11099381

[11] Yang H, et al. Genome‑wide pan‑GPCR cell libraries accelerate drug discovery. Acta Pharm Sin B. 2024;14(10):4296–311. 10.1016/j.apsb.2024.06.023. 39525595 10.1016/j.apsb.2024.06.023 PMC11544303

[12] Pándy‑Szekeres G, et al. GPCRdb in 2023: state‑specific structure models using AlphaFold2 and new ligand resources. Nucleic Acids Res. 2023;51(D1):D395–402. 10.1093/nar/gkac1013. 36395823 10.1093/nar/gkac1013 PMC9825476

[13] Lorente JS, et al. GPCR drug discovery: new agents, targets and indications. Nat Rev Drug Discov. 2025;24(6):458–79. 10.1038/s41573-025-01139-y. 40033110 10.1038/s41573-025-01139-y

[14] Takahashi K, et al. Neurotensin receptor 1 signaling promotes pancreatic cancer progression. Mol Oncol. 2021;15(1):151–66. 10.1002/1878-0261.12815. 33034134 10.1002/1878-0261.12815 PMC7782081

[15] Bugni JM, et al. The neurotensin receptor‑1 promotes tumor development in a sporadic but not an inflammation‑associated mouse model of colon cancer. Int J Cancer. 2012;130(8):1798–805. 10.1002/ijc.26208. 21630261 10.1002/ijc.26208 PMC3288327

[16] Reinecke M. Neurotensin. Immunohistochemical localization in central and peripheral nervous system and in endocrine cells and its functional role as neurotransmitter and endocrine hormone. Prog Histochem Cytochem. 1985;16(1):1–172. 2859633

[17] Baca I, et al. Effect of neurotensin on exocrine pancreatic secretion in dogs. Digestion. 1982;23(3):174–83. 10.1159/000198725. 7106418 10.1159/000198725

[18] Andersson S, et al. Inhibition of gastric and intestinal motor activity in dogs by (Gln4) neurotensin. Acta Physiol Scand. 1977;100(2):231–5. 10.1111/j.1748-1716.1977.tb05941.x. 888712 10.1111/j.1748-1716.1977.tb05941.x

[19] Ehlers RA, et al. Neurotensin‑mediated activation of MAPK pathways and AP‑1 binding in the human pancreatic cancer cell line, MIA PaCa‑2. Biochem Biophys Res Commun. 2000;269(3):704–8. 10.1006/bbrc.2000.2335. 10720480 10.1006/bbrc.2000.2335

[20] Zhao D, et al. Rho GTPases as therapeutic targets for the treatment of inflammatory diseases. Expert Opin Ther Targets. 2003;7(5):583–92. 10.1517/14728222.7.5.583. 14498821 10.1517/14728222.7.5.583

[21] Leyton J, et al. Neurotensin causes tyrosine phosphorylation of focal adhesion kinase in lung cancer cells. Eur J Pharmacol. 2002;442(3):179–86. 10.1016/S0014-2999(02)01539-X. 12065070 10.1016/s0014-2999(02)01539-x

[22] Zygulska AL, et al. Changes in plasma levels of cholecystokinin, neurotensin, VIP and PYY in gastric and colorectal cancer—preliminary results. Peptides. 2019;122:170148. 10.1016/j.peptides.2019.170148. 31541684 10.1016/j.peptides.2019.170148

[23] Wang JG, et al. Pancreatic cancer bears overexpression of neurotensin and neurotensin receptor subtype‑1 and SR 48692 counteracts neurotensin induced cell proliferation in human pancreatic ductal carcinoma cell line PANC‑1. Neuropeptides. 2011;45(2):151–6. 10.1016/j.npep.2011.01.002. 21272935 10.1016/j.npep.2011.01.002

[24] Qiu S, et al. A review of the role of neurotensin and its receptors in colorectal cancer. Gastroenterol Res Pract. 2017;2017:6456257. 10.1155/2017/6456257. 28316623 10.1155/2017/6456257 PMC5339424

[25] Moody TW, et al. SR48692 is a neurotensin receptor antagonist which inhibits the growth of small cell lung cancer cells. Peptides. 2001;22(1):109–15. 10.1016/S0196-9781(00)00362-4. 11179604 10.1016/s0196-9781(00)00362-4

[26] Souazé F, et al. Neurotensin receptor 1 gene activation by the Tcf/β‑catenin pathway is an early event in human colonic adenomas. Carcinogenesis. 2006;27(4):708–16. 10.1093/carcin/bgi269. 16299383 10.1093/carcin/bgi269

[27] Younes M, et al. Neurotensin (NTS) and its receptor (NTSR1) causes EGFR, HER2 and HER3 over‑expression and their autocrine/paracrine activation in lung tumors, confirming responsiveness to erlotinib. Oncotarget. 2014;5(18):8252–69. 10.18632/oncotarget.1633. 25249545 10.18632/oncotarget.1633 PMC4226681

[28] Nikolaou S, et al. The role of neurotensin and its receptors in non‑gastrointestinal cancers: a review. Cell Commun Signal. 2020;18(1):68. 10.1186/s12964-020-00569-y. 32336282 10.1186/s12964-020-00569-y PMC7183616

[29] Reubi JC, et al. Neurotensin receptors: a new marker for human ductal pancreatic adenocarcinoma. Gut. 1998;42(4):546–50. 10.1136/gut.42.4.546. 9616318 10.1136/gut.42.4.546 PMC1727058

[30] Dupouy S, et al. Activation of EGFR, HER2 and HER3 by neurotensin/neurotensin receptor 1 renders breast tumors aggressive yet highly responsive to lapatinib and metformin in mice. Oncotarget. 2014;5(18):8235–51. 10.18632/oncotarget.1632. 25249538 10.18632/oncotarget.1632 PMC4226680

[31] DaSilva JO, et al. Neuroendocrine‑derived peptides promote prostate cancer cell survival through activation of IGF‑1R signaling. Prostate. 2013;73(8):801–12. 10.1002/pros.22624. 23192379 10.1002/pros.22624 PMC4085781

[32] Valerie NC, et al. Inhibition of neurotensin receptor 1 selectively sensitizes prostate cancer to ionizing radiation. Cancer Res. 2011;71(21):6817–26. 10.1158/0008-5472.CAN-11-1646. 21903767 10.1158/0008-5472.CAN-11-1646

[33] Müller KM, et al. Role of protein kinase C and epidermal growth factor receptor signalling in growth stimulation by neurotensin in colon carcinoma cells. BMC Cancer. 2011;11:421. 10.1186/1471-2407-11-421. 21961726 10.1186/1471-2407-11-421 PMC3196723

[34] Guha S, et al. Neurotensin stimulates protein kinase C-dependent mitogenic signaling in human pancreatic carcinoma cell line PANC-1. Cancer Res. 2003;63(10):2379–87. 12750255

[35] Hassan S, et al. Involvement of MAP‑kinase, PI3‑kinase and EGF‑receptor in the stimulatory effect of neurotensin on DNA synthesis in PC3 cells. Regul Pept. 2004;120(1–3):155–66. 10.1016/j.regpep.2004.03.004. 15177934 10.1016/j.regpep.2004.03.004

[36] Zhang Y, et al. Neurotensin receptor1 antagonist SR48692 reduces proliferation by inducing apoptosis and cell cycle arrest in melanoma cells. Mol Cell Biochem. 2014;389(1–2):1–8. 10.1007/s11010-013-1920-3. 24357116 10.1007/s11010-013-1920-3

[37] Slosky LM, et al. β‑Arrestin‑biased allosteric modulator of NTSR1 selectively attenuates addictive behaviors. Cell. 2020;181(6):1364-1379.e14. 10.1016/j.cell.2020.04.053. 32470395 10.1016/j.cell.2020.04.053 PMC7466280

[38] Moore MN, et al. Designing allosteric modulators to change GPCR G protein subtype selectivity. Nature. 2025;648(8092):229–38. 10.1038/s41586-025-09643-2. 41125894 10.1038/s41586-025-09643-2 PMC12675282

[39] Gu A, et al. Patient‑derived xenograft model in cancer: establishment and applications. MedComm. 2025;6(2):e70059. 10.1002/mco2.70059. 39830019 10.1002/mco2.70059 PMC11742426

[40] Tong S, et al. Pharmacokinetics and bioavailability of liensinine in mouse blood by UPLC‑MS/MS. Acta Chromatogr. 2021;33(4):333–7. 10.1556/1326.2020.00847.

